# Connectome-based predictive modeling of smoking severity using individualized structural covariance network in smokers

**DOI:** 10.3389/fnins.2023.1227422

**Published:** 2023-07-21

**Authors:** Weijian Wang, Yimeng Kang, Xiaoyu Niu, Zanxia Zhang, Shujian Li, Xinyu Gao, Mengzhe Zhang, Jingliang Cheng, Yong Zhang

**Affiliations:** Department of Magnetic Resonance Imaging, The First Affiliated Hospital of Zhengzhou University, Zhengzhou, China

**Keywords:** connectome-based predictive modeling, structural covariance, nicotine addiction, smoking severity, machine learning

## Abstract

**Introduction:**

Abnormal interactions among distributed brain systems are implicated in the mechanisms of nicotine addiction. However, the relationship between the structural covariance network, a measure of brain connectivity, and smoking severity remains unclear. To fill this gap, this study aimed to investigate the relationship between structural covariance network and smoking severity in smokers.

**Methods:**

A total of 101 male smokers and 51 male non-smokers were recruited, and they underwent a T1-weighted anatomical image scan. First, an individualized structural covariance network was derived via a jackknife-bias estimation procedure for each participant. Then, a data-driven machine learning method called connectome-based predictive modeling (CPM) was conducted to infer smoking severity measured with Fagerström Test for Nicotine Dependence (FTND) scores using an individualized structural covariance network. The performance of CPM was evaluated using the leave-one-out cross-validation and a permutation testing.

**Results:**

As a result, CPM identified the smoking severity-related structural covariance network, as indicated by a significant correlation between predicted and actual FTND scores (r = 0.23, permutation *p* = 0.020). Identified networks comprised of edges mainly located between the subcortical–cerebellum network and networks including the frontoparietal default model and motor and visual networks.

**Discussion:**

These results identified smoking severity-related structural covariance networks and provided a new insight into the neural underpinnings of smoking severity.

## Introduction

Nicotine addiction or smoking, characterized by compulsive tobacco seeking and smoking, is one of the leading causes of preventable disease worldwide, contributing to cancer and respiratory and cardiovascular diseases (Branstetter et al., 2016; Shen et al., 2018). Every year, more than 5 million people die of tobacco use (Wen et al., 2021). It is of great clinical significance to investigate the underlying neural substrates of nicotine addiction. However, the underlying mechanisms of smoking are still unclear.

In recent years, studies with modern neuroimaging have identified that abnormal interactions among distributed brain regions are implicated in the mechanisms of nicotine addiction (Fedota and Stein, 2015). In neuroimaging studies, the interaction between brain regions is usually measured with synchronized low-frequency fluctuations of the blood oxygen level-dependent (BOLD) signal between brain regions in the absence of explicit task, termed as resting-state functional connectivity (FC) (Biswal et al., 1995; Molecular Psychiatry). Altered FC among large-scale brain networks is well documented in smokers. For example, relative to non-smokers, chronic smokers show reduced FC within frontal–parietal executive control networks (Weiland et al., 2015), and connections within this network are predictive of smoking status (Pariyadath et al., 2014). Structural and functional deficits within frontostriatal circuits are also reported in smokers (Yuan et al., 2016), where the striatum mainly regulates the rewarding effect of smoking (Barrett et al., 2004) and motivation to smoke (Le Foll et al., 2014). In addition to functional interaction, brain regions also exhibit structural interactions, such as structural covariance. As another brain connectivity metric, structural covariance measures gray matter morphological similarity (measured with correlation) between brain regions reflecting synchronized maturation (Alexander-Bloch et al., 2013; Yun et al., 2015). Compared with FC, structural covariance represents more stable and highly heritable brain connectivity features (Lerch et al., 2006; Evans, 2013). As a complementary metric of brain connectivity, structural covariance provides a specific and distinctive measurement of network-level brain features (Subirà et al., 2016). Brain disorders are found to be accompanied by a rearranged architecture of the structural covariance network (Mitelman et al., 2005; Alexander-Bloch et al., 2013; Han et al., 2022a,b; Xue et al., 2023). However, to the best of our knowledge, there is no study investigating the association between structural covariance network and smoking severity in nicotine addiction.

Recently, a machine learning method called connectome-based predictive modeling (CPM) is proposed to predict individual behavior from whole-brain connectivity data (“connectomes”) (Finn et al., 2015; Shen et al., 2017). Unlike the traditional brain-behavior models such as correlation or regression models, CPM avoids over-fitting and increases the likelihood of generalization in novel samples. This approach is used to identify connectome fingerprints of specific behaviors, showing potential to identify novel treatment targets (Finn et al., 2015; Shen et al., 2017). To date, CPM has been used to identify connectome fingerprints of IQ (Finn et al., 2015), individual anxiety (Wang et al., 2021), and creativity anxiety (Ren et al., 2021). In addition, CPM identifies connectome fingerprints of carving intensities in Internet gaming disorder (Zhou et al., 2022), dissociable neural substrates of opioid and cocaine use (Lichenstein et al., 2021), and connectome fingerprints of cocaine abstinence (Yip et al., 2019).

In this study, we aimed to identify the structural covariance network predictive of smoking severity in smokers with CPM. A total of 101 smokers and 51 non-smokers were recruited, and they underwent T1-weighted anatomical image scans. First, we obtained an individualized structural covariance network for each participant via a jackknife-bias estimation procedure (Das et al., 2018; Han et al., 2022a,b). Then, CPM was conducted to infer smoking severity measured with Fagerström Test for Nicotine Dependence (FTND) scores (Heatherton et al., 1991) using the individualized structural covariance network. We expected that CPM could significantly predict FTND scores using the individualized structural covariance network over chance.

## Methods

### Samples

This study was approved by the Research Ethics Committee of the First Affiliated Hospital of Zhengzhou University. All study procedures were performed in accordance with the 1975 Declaration of Helsinki, and written informed consent was obtained from all participants before the experiment.

A total of 101 smokers and 51 non-smokers were recruited through online platforms and advertisements. All the subjects were right-handed male smokers. The smokers met the DSM-IV criteria for nicotine dependence, smoked at least once daily in the past 2 years, and had no period of smoking abstinence longer than 3 months (Shen et al., 2018). The Fagerström Test for Nicotine Dependence (FTND) was used to measure nicotine dependence (Heatherton et al., 1991). Non-smokers are those who did not currently smoke and had no history of consumption of cigarettes (or nicotine products). In addition, all the participants must meet the following inclusion criteria: (1) physical and neuropsychiatric diseases; (2) currently using or a history of psychotropic medications; (3) other current drug abuse (except nicotine); and (4) contraindications for MRI scanning.

### Data acquisition

Three-dimensional high-resolution T1-weighted sagittal images were obtained with 3D magnetization prepared rapid gradient echo (3D-MPRAGE) on 3-Tsela German Siemens Prisma. The scanning parameters were as follows: repetition time = 2,000 ms, voxel size = 1 mm^3^, inversion time = 900 ms, echo time = 2.06 ms, flip angle = 9 degrees, FOV = 256 × 256 mm^2^, slices = 192, and slice thickness = 1.0 mm.

### Voxel-based morphometry analysis

Gray matter volume (GMV) was measured using a voxel-based morphometry analysis (VBM) (Ashburner and Friston, 2000). This procedure was conducted following the standard pipeline of the CAT12 toolbox (http://dbm.neuro.uni-jena.de/cat12/): Structural images were first segmented into the gray matter, white matter, and cerebrospinal fluid, normalized into Montreal Neurological Institute space and resampled to 1.5 mm^3^. Finally, the gray matter maps were smoothed using 6 mm full width at half maximum Gaussian kernel (FWHM) (Ashburner, 2009; Han et al., 2021). The total intracranial volume (TIV) and image quality rating (IQR) were calculated (Brown et al., 2019; Han et al., 2022a,b, 2023).

### Construction of individualized structural covariance network

Following previous studies (Das et al., 2018; Han et al., 2022a,b), an individualized structural covariance network was constructed using the following steps. First, a group-level N × N (N, the number of brain regions) structural covariance network was constructed (SCN) for smokers. Specifically, mean GMV values of brain regions defined in the automated anatomical labeling atlas (AAL) were extracted, and pairwise Pearson's correlations between them were calculated. Thus, a group-level 116 × 116 SCN was obtained. Second, for each subject, individualized SCN was derived via a jackknife-bias estimation procedure that determined an individual's contribution to the group-level SCN (Miller, 1974; Das et al., 2018).

### Connectome-based predictive modeling

We constructed a CPM to predict FTND scores from individualized SCN in smokers. In brief, in the training dataset, structural covariance edges and FTND scores were correlated using Pearson's correlation. The whole network was divided into positive and negative predictive networks according to the sign of significant Pearson's correlation coefficients (*p* < 0.01). Then, the sum of significant edges in each network was obtained and entered into a linear regression model to establish the linear relationship between the edges and FTND scores. The resultant polynomial coefficients including slope and intercept were applied to the test dataset to predict FTND scores. In this study, leave-one-out cross-validation (LOOCV) was adopted. The performance of CPM was assessed by calculating Pearson's correlation between the predicted FTND scores and true ones.

To assess whether the performance of CPM (Pearson's correlation coefficients between the predicted FTND scores and true ones) was significantly higher than chance, a permutation testing was performed (5,000 times). In each run, FTND scores and connections were randomly shuffled, and CPM was rerun with the shuffled data. The significance was defined as the proportion of sampled permutations that were greater or equal to the true prediction correlation (Shen et al., 2017).

### Sensitivity analysis

We also explored whether our results were biased by confounding factors including TIV or IQR. To this end, Pearson's correlation coefficients between predicted FTND scores and TIV or IQR were calculated.

## Results

### Demographics and clinical characteristics

Demographics and clinical characteristics are included in Table 1. There were no significant differences in terms of age, education level, and IQR between smokers and non-smokers.

**Table 1:** Demographics and clinical characteristics.

	**Smokers (*N* = 101)**	**Non-smokers (*N* = 52)**	***p***
Male, No. (%)	100%	100%	-
Age, mean (SD)	35.01 (7.26)	39.94 (7.60)	0.103^a^
Educational level, mean (SD), y	14.88 (1.97)	15.37 (2.65)	0.204^a^
FTND	5.92 (3.51)	-	-
Smoking years	19.37 (3.18)	-	-
Cigarettes smoked per day	15.56 (7.12)	-	-
IQR	2.06 (0.11)	2.04 (0.09)	0.323^a^

FTND, Fagerström Test for Nicotine Dependence; IQR, Image Quality Rating;

a two sample *t-*test.

### Performance of CPM

**Figure 1:**
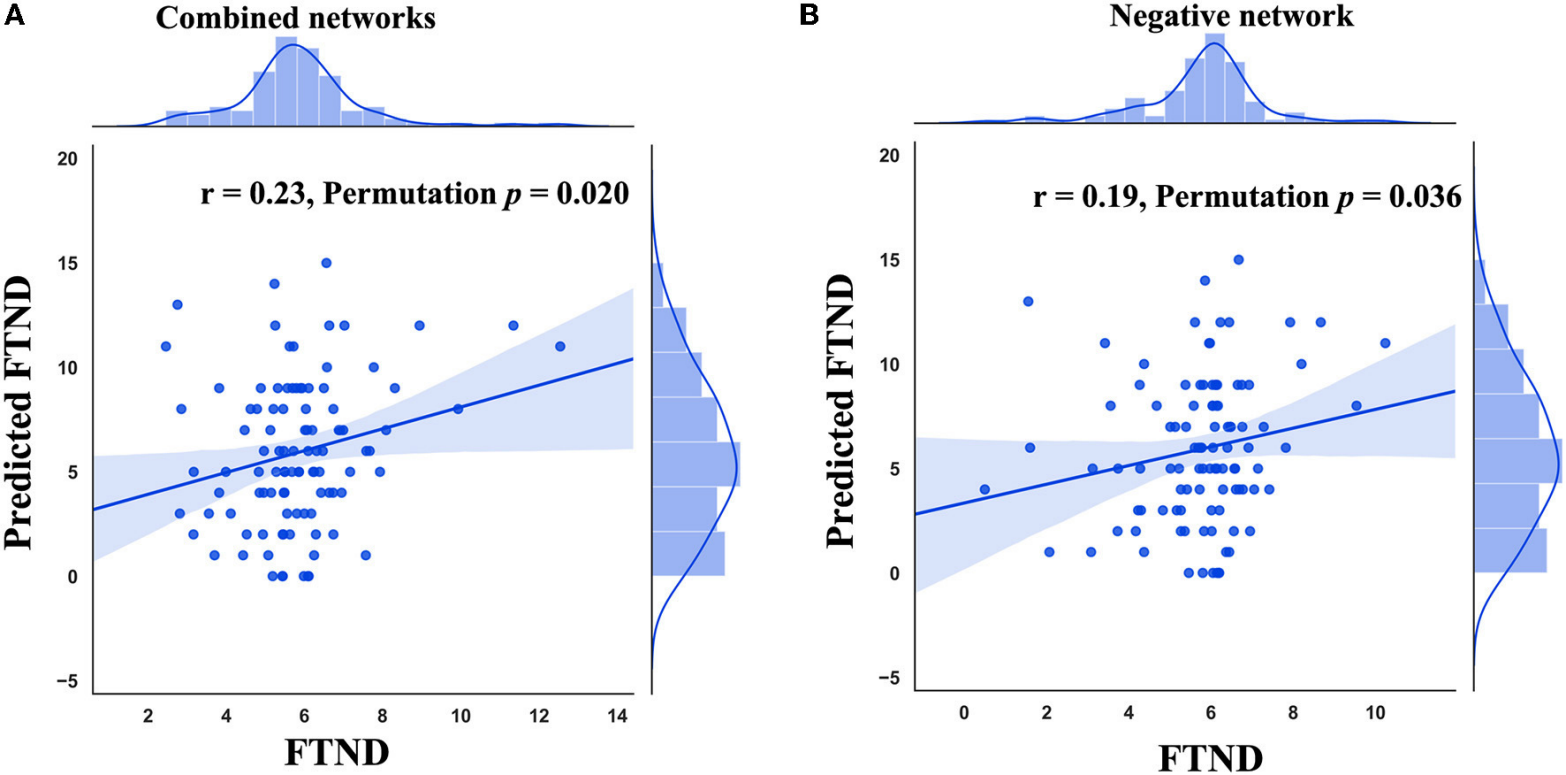
Performance of connectome-based predictive modeling (CPM). **(A)** The correlation between predicted FTND scores using combined networks and true ones. **(B)** The correlation between predicted FTND scores using the negative network and true ones. FTND, Fagerström Test for Nicotine Dependence.

Combining positive and negative structural covariance networks, CPM significantly predicted the FTND scores (r = 0.23, permutation *p* = 0.020, Figure 1A). We further investigated whether a negative or positive network alone could predict the FTND scores. The results showed that a negative network could significantly predict FTND scores (r = 0.19, permutation *p* = 0.036, Figure 1B) while a positive network could not (permutation *p* > 0.05).

### Smoking severity-related network anatomy

**Figure 2:**
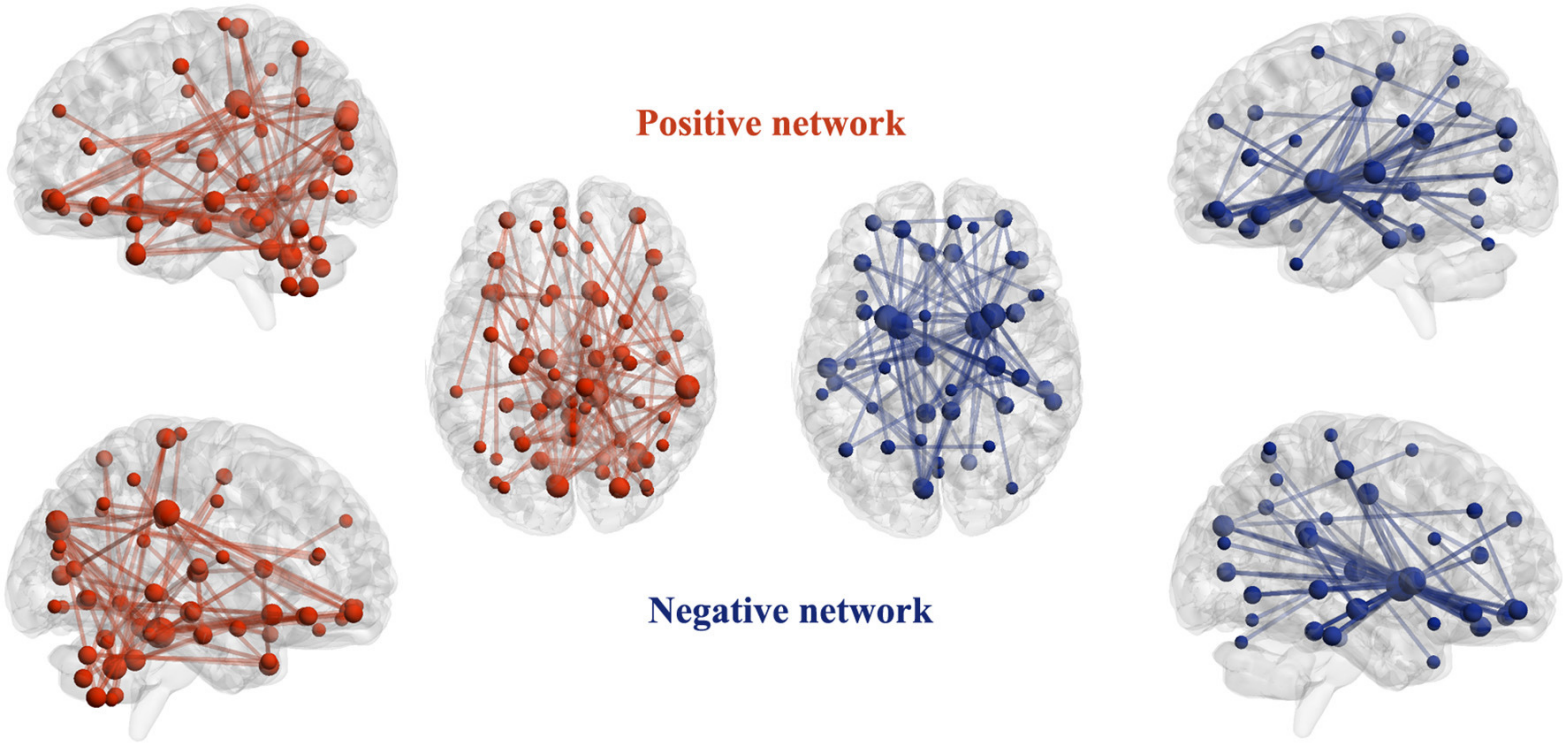
Identified positive and negative networks. For the positive network, increased structural covariance edges predict more smoking severity measured with Fagerström Test for Nicotine Dependence scores. For the negative network, decreased structural covariance edges predict more smoking severity measured with Fagerström Test for Nicotine Dependence scores. The size of nodes is proportional to the number of edges attached to them (degree).

The identified positive and negative networks are presented in Figure 2. The positive network included 95 edges, and the negative network included 103 edges. The node size in Figure 2 was proportional to the degree. Highest-degree nodes in the positive network were mainly located in the cerebellum, thalamus, hippocampus, and superior occipital gyrus, and those in the negative network were mainly located in the striatum, hippocampus, cingulate gyrus, and orbitofrontal cortex. The top 10 nodes with the highest degree in the positive and negative networks are listed in Table 2.

**Table 2:** Top ten nodes with the highest degree in positive and negative network.

**Network**	**Nodes**	**Degree**	**Network**	**Nodes**	**Degree**
Positive	Right superior cerebellum	20	Negative	Right pallidum	27
	Right supramarginal gyrus	11		Left pallidum	22
	Left cuneus	8		Left putamen	17
	Vermis	8		Right putamen	17
	Right superior occipital gyrus	6		Right hippocampus	7
	Left superior cerebellum	6		Left superior temporal gyrus	7
	Right olfactory	5		Left middle cingulate gyrus	6
	Left hippocampus	5		Left posterior cingulate gyrus	6
	Left thalamus	5		Left cuneus	6
	Vermis	5		Right orbitofrontal cortex	5

**Figure 3:**
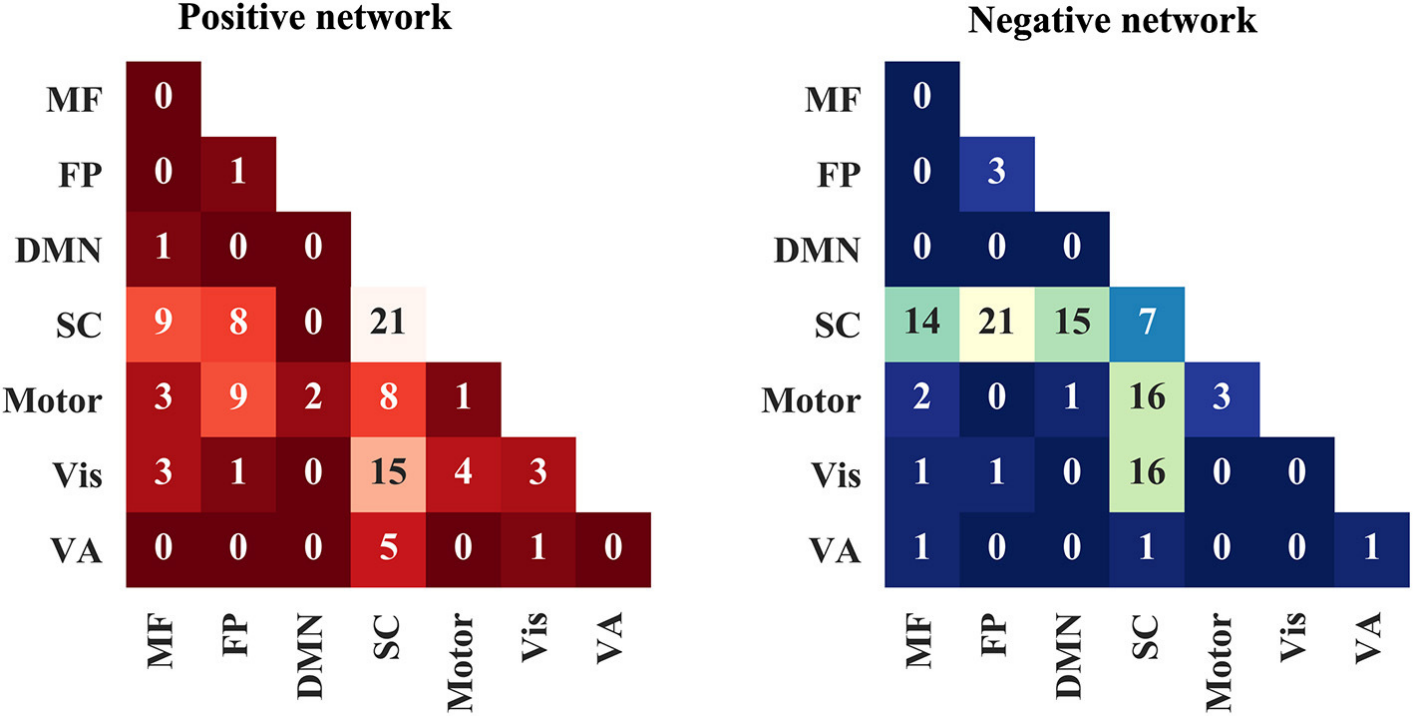
Positive and negative networks are summarized into within and between canonical neural networks. MF, medial frontal network; FP, frontoparietal network; DMN, default model network; SC, subcortical-cerebellum network; VA, visual association network.

Next, the edges in positive and negative networks were summarized into within and between canonical neural networks as defined in previous studies (Finn et al., 2015; Han et al., 2022a,b). The number of edges between and within the networks are presented in Figure 3. The edges in the positive network were mainly distributed within the subcortical–cerebellum network between the subcortical–cerebellum network and the visual network. The edges in the negative network were mainly located between the subcortical–cerebellum network and those including the frontoparietal network, default model network (DMN), and motor and visual network.

### Sensitivity analysis

We did not observe any significant correlation between factors including TIV and IQR and predicted FTND scores (all *p*-values > 0.05), excluding their effects on the performance of CPM.

## Discussion

In this study, we successfully predicted smoking severity from the individualized structural covariance network using CPM and identified smoking severity-related networks. The combined and negative network could significantly predict the FTND scores, suggesting their close association with smoking severity. The identified negative network mainly comprised of structural covariance edges between the subcortical–cerebellum network and the networks including frontoparietal and motor and visual. In this network, the putamen, pallidun, hippocampus, cingulate gyrus, and orbitofrontal cortex had the highest degree reflecting their vital roles in this network. These results elucidate the neuronal substrates of smoking severity establishing the connection between structural covariance network and smoking severity for the first time.

Our results reveal that individualized structural covariance across multiple brain networks is predictive of smoking severity. With CPM, previous studies have also identified severity-related functional networks, such as nicotine addiction, cocaine, Internet gaming disorder, and opioid use disorder (Yip et al., 2019; Lichenstein et al., 2021; Lin et al., 2022; Zhou et al., 2022). As a state feature, functional connectivity may oscillate across different states and is susceptible to many confounding factors (Evans, 2013; Gorgolewski et al., 2013). In this study, we identified severity-related structural covariance networks in smokers. Compared with functional connectivity, structural covariance measures brain connectivity on a larger time scale and is hypothesized to represent trait-like connectivity features (Subirà et al., 2016). In spite of substantial concordance between functional connectivity and structural covariance, they are different and complementary metrics of brain connectivity (Subirà et al., 2016). However, to the best of our knowledge, there are only two studies investigating altered structural covariance in addiction-related disorders, namely Internet gaming disorder and alcohol dependence (Chen et al., 2021; Ottino-González and Garavan, 2022). The former elucidates the association between decreased structural covariance connections within DMN and addiction severity in Internet gaming disorder (Chen et al., 2021). The latter investigates altered graph-theoretic metrics of the structural covariance network in adults with alcohol dependence and heavy-drinking adolescents and suggests that the structural covariance network profile can be an early marker of alcohol dependence in adults (Ottino-González and Garavan, 2022). However, both of them investigate structural covariance network aberrance at the group level. With the help of a newly proposed method, for the first time, we investigated the association between structural covariance network and smoking severity at the individual level. We found that the combined and negative networks could significantly predict the FTND score while the positive network could not. These results are consistent with the study by Lin et al. (2022) investigating functional connectivity networks underpinning smoking severity. These results suggest that smoking severity is associated with impaired coordination among brain networks.

Our results revealed that decreased structural covariance connections between the subcortical–cerebellum network and other networks played vital roles in the mechanisms of nicotine addiction. The subcortical–cerebellum network defined in this study contains regions, such as the hippocampus, amygdala, striatum, thalamus, orbitofrontal gyrus, anterior insula, and cerebellum (Finn et al., 2015). Among these brain regions, the pallidum, putamen, hippocampus, and orbitofrontal gyrus had the highest degree indicating their important roles in the identified negative network. All these regions are implicated in the brain reward system that is consistently hypothesized to underlay the mechanism of addiction (Hyman et al., 2006; Haber and Knutson, 2010). For example, the putamen plays a vital role in the development of nicotine addiction due to the high concentration of nicotinic acetylcholine receptors which makes it a potential target for nicotine (Das et al., 2012). In the putamen, hyperactivity, when exposed to environmental cues triggering craving and gray matter volume abnormalities, is reported in smokers compared to non-smokers (Das et al., 2012; Pan et al., 2013; Franklin et al., 2014). In addition, gray matter volume is significantly correlated with pack years, suggesting it is a potential biomarker of the cumulative effect of smoking (Bu et al., 2016). In the hippocampus, preclinical studies consistently suggest that its function is enhanced by initial exposure to drugs, and the synaptic alterations by stimulants can facilitate the learning of drug-associated memories and eventual addiction to the drug (Avchalumov and Mandyam, 2021). Recent evidence indicates the involvement of the cerebellum in addictive behavior (Kühn et al., 2012). Tissue volume loss in the cerebellum is reported in smokers (Kühn et al., 2012), and the decreased connections between the cerebellum and DMN are also reported in smokers relative to non-smokers (Wetherill et al., 2015; Shen et al., 2018). In the cerebellum, repeated drug exposure enhances the susceptibility of its connections with the frontal gyrus that is related to impaired executive control of the prefrontal cortex on drug-seeking behavior (Miquel et al., 2009). In accordance with these findings, our results further underscore the vital role of the subcortical–cerebellum network in the smoking severity.

Other networks including frontoparietal, DMN, and motor and visual networks are also implicated in the mechanisms of addiction severity. The frontoparietal network is implicated in representing and maintaining goals during motivated behavior (Koechlin and Hyafil, 2007; Badre, 2008) and has a prominent role in inhibitory control of impulsive responses (Jentsch and Taylor, 1999). People with substance dependence and behavioral addictions show hypoactivation during motor response inhibition tasks and cognitive self-regulation (Luijten et al., 2014). In addition, its weak connections with the striatal system during a response inhibition task are associated with greater dependence severity in alcoholics (Courtney et al., 2013). The dysfunction of the frontoparietal network contributes to the transition from voluntary/goal to a more habitual drug-seeking behavior (Everitt and Robbins, 2005). The dysfunction of DMN and the abnormal interactions between DMN and other networks are hypothesized to contribute to craving and relapse in substance abuse (Zhang and Volkow, 2019). For example, as a central component of the valuation system of the brain, the medial prefrontal cortex is involved in the dysregulation of the reward and motivation circuits in addiction (Volkow et al., 2003; Bartra et al., 2013). The weaker connectivity between the putamen and Medial prefrontal cortex during an inhibition task is associated with greater alcohol dependence severity (Courtney et al., 2013). Altered connections between the midline core DMN and subcortical regions, such as the amygdala and the striatum, are responsible for the cognitive, emotional, and reward-related dysregulation in substance abuse (Koob and Volkow, 2016). In addition, connections between the subcortical–cerebellum network and the motor and visual networks are also found to be associated with smoking severity. Exposure to nicotine/cannabis can result in long-lasting dysregulation of somatosensory processing and less motor cortical plasticity (Smolka et al., 2006). In addition, previous studies have revealed that the activations of motor- and vision-related brain regions in response-related cues are correlated with smoking severity (Yalachkov et al., 2009) and are predictive of relapse (Kosten et al., 2006). In accordance with these findings, our results suggest that decreased connections between the subcortical–cerebellum network and these other networks underlay the neural underpinnings of smoking severity.

Several limitations in this study should be mentioned. First, our findings are drawn from one dataset with a moderate sample size, and whether these findings can be reproduced in other independent datasets with a larger sample size needs to be assessed. Second, only male smokers are included in this study, and future studies could investigate whether our findings hold true in female smokers. Third, we investigated the smoking severity-related structural covariance network using cross-sectional data, and whether and how the identified networks progress with increased severity need to be elucidated using longitudinal data in future.

## Data availability statement

The raw data supporting the conclusions of this article will be made available by the authors, without undue reservation.

## Ethics statement

The studies involving human participants were reviewed and approved by the Research Ethics Committee of the First Affiliated Hospital of the Zhengzhou University. The patients/participants provided their written informed consent to participate in this study.

## Author contributions

Study concept and design: WW and YZ. Data acquisition: YK, XN, and ZZ. Data analysis and interpretation of data: WW, SL, and YK. Drafting of the initial manuscript: WW, YK, MZ, XG, JC, and YZ. All authors contributed to the article and approved the submitted version.
